# Altitude and child development: A cross‐sectional comparative study of children in urbanized low‐altitude and rural high‐altitude settings in Kyrgyzstan

**DOI:** 10.14814/phy2.71132

**Published:** 2026-10-12

**Authors:** Adam Bøcker Toft Olsen, Elvira Isaeva, Madina Umetalieva, Nargiza Osmonbaeva, Sofie Valborg Wright, Lea Deniz Lund Serefoglu, Freya Natasha Kretzchmar, Trisse Dyrløv Høegh, Jesper Kjærgaard, Kim Gjerum Nielsen, Talant Sooronbaev, Anja Poulsen

**Affiliations:** ^1^ Faculty of Health and Medical Sciences University of Copenhagen Copenhagen Denmark; ^2^ National Centre of Maternity and Childhood Care (NCMCC) Bishkek Kyrgyz Republic; ^3^ International Higher School of Medicine Bishkek Kyrgyz Republic; ^4^ University College Copenhagen Copenhagen Denmark; ^5^ Department of Pediatrics and Adolescent Medicine, Juliane Marie Centre Copenhagen University Hospital, Rigshospitalet Copenhagen Denmark; ^6^ National Centre of Cardiology and Internal Medicine Named After Academician M. Mirrakhimov Bishkek Kyrgyz Republic

**Keywords:** altitude, Asia, Central, child development, LMIC, respiratory function tests

## Abstract

Childhood malnutrition remains a global challenge; Kyrgyzstan has reduced it, though disparities persist. We hypothesized that developmental differences between lowland and highland children reflect altitude adaptation, not malnutrition alone, obscuring monitoring. This study compared children from Naryn (2400 m) to Bishkek (800 m), examining respiratory, hematological, and anthropometric development. We conducted a cross‐sectional study among healthy Kyrgyz schoolchildren aged 10–12 from Naryn and Bishkek. Measurements included anthropometrics, spirometry, hemoglobin, hand‐grip strength, and vital signs. Linear regression controlled for socioeconomic, maternal, nutritional, and healthcare factors. Compared to Naryn (*n* = 143), Bishkek children (*n* = 123) were taller (2.7 cm, *p* = 0.006), heavier (2.7 kg, *p* = 0.008), with larger waist (2.7 cm, *p* = 0.007) and mid‐upper arm circumferences (0.7 cm, *p* = 0.023), lower hemoglobin (−14.4 g/L, *p* < 0.001), higher oxygen saturation (2.1%, *p* < 0.001), weaker hand‐grip strength (−1.3 kgf, *p* = 0.013), and higher HAZ (0.5, *p* < 0.001). No differences existed in BMI or BAZ. Naryn males were shorter than females (−3.60 cm, *p* = 0.024). Bishkek children had lower FEV1 (−0.4, *p* = 0.006) and FVC *z*‐scores (−0.48, *p* = 0.003). High‐altitude Kyrgyz children showed developmental and respiratory patterns suggesting physiological adaptation, though malnutrition cannot be excluded. Lower birthweight partially explained reduced height but not lung function or male‐specific growth, highlighting the need for altitude‐specific growth and respiratory references and continued research in pediatric high‐altitude populations.

## Graphical abstract

The graphic abstract was created by Olsen, A. B. T. using Inkscape. The image in the right “Naryn” pane was privately sourced. The image in the left “Bishkek” pane is sourced from Wikipedia: Aitenir, 2017. “Bishkek's City business center”. https://upload.wikimedia.org/wikipedia/commons/4/42/Bishkek_City%27s_business_center.jpg. CC0, via Wikimedia Commons.

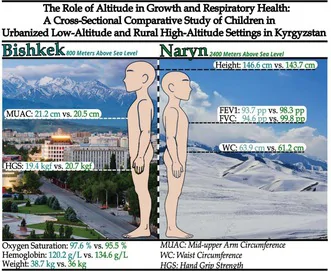

## 1 INTRODUCTION

Childhood malnutrition remains a major global health challenge. According to the World Health Organization (WHO), approximately 149 million children under the age of five are affected by stunting worldwide (WHO, 2024). Stunting reflects chronic undernutrition and is associated with poor socioeconomic conditions, inadequate maternal health, insufficient dietary intake, recurrent infections, and impaired physical and cognitive development (WHO, 2024).

Kyrgyzstan, a predominantly mountainous country in Central Asia, has achieved substantial progress in reducing childhood malnutrition over the past two decades (National Statistical Committee of the Kyrgyz Republic (NSC) et al., 2013; National Statistical Committee of the Kyrgyz Republic & UNICEF, 2024; Wigle et al., 2020). Between 1997 and 2014, the prevalence of stunting declined markedly from 36.2% to 12.9% (UNICEF, 2025). Despite this remarkable improvement, persistent regional disparities remain, particularly in high‐altitude areas (Wigle et al., 2020).

Approximately 94% of the country's territory is mountainous, with elevations ranging from 400 to over 7000 meters above sea level. The majority of its nearly seven million inhabitants reside in valleys and lowland areas, while remote high‐altitude regions are characterized by rural settlement patterns, limited infrastructure, and reduced access to healthcare services. Such geographic and socioeconomic divides may contribute to unequal nutritional outcomes across altitude and rural–urban settings (O'Brien et al., 2005).

Children living at high altitude show distinct physiological adaptations, including altered hemoglobin, lung function, strength, and cardiovascular fitness (Al‐Hashem, 2006; Fiori et al., 2000; Frisancho, 2013; Santos et al., 2020; Weitz et al., 2002; Weitz & Garruto, 2015). Traditionally, stunting in these populations has been attributed to environmental factors such as poverty, food insecurity, and limited healthcare access. However, recent evidence suggests that altitude exposure itself may impact linear growth, even after controlling for confounders (Baye & Hirvonen, 2020; Shively & Schmiess, 2021). Furthermore, recent evidence suggests that focusing solely on linear growth may provide an incomplete picture of child development in high‐altitude regions, as altitude‐related physiological changes could influence various aspects of physical development and functional capacity (Leroy & Frongillo, 2019; Scheffler et al., 2020). Kyrgyzstan has implemented numerous initiatives to combat child malnutrition (Akseer et al., 2018; Lundeen et al., 2019; Wigle et al., 2020) and regularly monitors linear growth and other parameters (Ministry of Health of the Kyrgyz Republic et al., 2022). This makes the country an ideal setting to examine whether developmental differences between highland and lowland children persist despite improved nutritional interventions. Currently, few studies have assessed multiple developmental outcomes in the same sample, making it difficult to disentangle physiological effects of altitude from environmental influences.

This exploratory study investigates developmental differences between highland and lowland school children in Kyrgyzstan across multiple functional and anthropometric measures. We hypothesize that children from high‐altitude regions will demonstrate distinct patterns of physical development compared to their lowland counterparts. By adjusting for socioeconomic and environmental factors, we aim to examine the contribution of altitude exposure to child development.

## 2 MATERIALS AND METHODS

### 2.1 Study design and setting

The study was structured as an observational cross‐sectional comparative study conducted in Kyrgyzstan between February and June 2025. The study compared healthy schoolchildren residing at high altitude (2400 meters above sea level (MASL)) and low altitude (800 MASL).

High‐altitude participants were recruited from the Naryn region, specifically from the rural villages of Ak‐Muz and Ozgorush (both located at 2400 MASL). Low‐altitude participants were recruited from School No. 96 in Bishkek, the capital of Kyrgyzstan (approximately 800 MASL).

### 2.2 Ethics

Ethical approval was granted by the Ethics Committee for Conducting Clinical Trials and Biomedical Research at the National center of Maternity and Childhood Care of the Ministry of Health and Social Development of the Kyrgyz Republic. February 7, 2025. No IRB approval number was provided. Signed ethical approval can be made available from the corresponding author upon reasonable request. The ethical execution of the study as well as the collection of signed informed consent was carried out in accordance with the Declaration of Helsinki of 1964.

### 2.3 Participants

Healthy schoolchildren aged 10–12 years were recruited from the participating schools, which were selected on a convenience sampling basis. Permission to sample participants from the two high‐altitude villages of Ak‐Muz and Ozgorush was granted by the local school district, and upon agreement with the local school directors. Permission to sample participants from the low‐altitude school of No. 96 in Bishkek was granted upon agreement with the Department of School Education under the Ministry of Education and Science of Kyrgyzstan.

### 2.4 Screening

Screening of parents and examinations of children were arranged in the following order. Classes comprising children aged 10–12 years were identified for inclusion. Parents of these children were invited to an informal meeting, where written informed consent was obtained. Subsequently, children were screened for eligibility, and parents were asked to complete a 20‐item questionnaire. Eligible children were then invited to undergo clinical examinations.

The eligibility criteria included fractional age of 10.00–12.99, >50% of life lived in the region, enrollment in the participating school, and parent signature of the informed consent form. Exclusion criteria were chronic health conditions such as asthma, diabetes, epilepsy, or thyroid disease. Additional exclusions included acute health conditions such as acute respiratory tract infection, pneumonia, or diarrhea. Children taking medications such as corticosteroids, bronchodilators, or diabetes medication were also excluded. Other exclusions were participation in other health intervention studies, parental non‐compliance, or inability to perform all the required tests.

### 2.5 Exposure and outcomes

We assessed nutritional status, functional parameters, and growth. Specifically, our research assessed height, height‐for‐age *z*‐scores (HAZ), weight, BMI, BMI‐for‐age *z*‐scores (BAZ), waist circumference (WC), mid‐upper arm circumference (MUAC), oxygen saturation, pulse rate, lung function (FEV_1_, FVC, and FEV_1_/FVC), hemoglobin levels, and hand grip strength (HGS).

Our study therefore consisted of the following exposure and outcomes:

Altitude (2400 MASL vs. 800 MASL) was defined as the primary exposure. The primary outcomes included anthropometric indicators of growth (height, weight, HAZ, and BAZ), lung function parameters (FEV_1_, FVC, and FEV_1_/FVC ratio), and hemoglobin concentrations. Secondary outcomes comprised MUAC, WC, HGS, SpO_2_, and pulse rate.

### 2.6 Questionnaire and variable definitions

The questionnaire consisted of 20 items grouped in six domains. These included: (1) household demographics and family structure, (2) early life factors including maternal characteristics, (3) nutritional practices and 24‐h dietary recall, (4) healthcare access and vaccination status, (5) socioeconomic indicators including income, housing, utilities, and water access, and (6) household smoking. The full questionnaire can be found in Data S1.

While key variables such as gender, age, height, weight, birth altitude, and birthweight were measured in conventional units, socioeconomic status was measured using household monthly income categories.

Dietary diversity was calculated as the total number of food groups consumed in 24‐h recall. Supplement count represented the number of dietary supplements used during child‐rearing, drawn from a predefined list including zinc, vitamin A, vitamin D, multivitamins, gulazyk, and omega‐3. Duration of supplementation was not captured. Vaccination count was the total number of completed vaccination time points. Healthcare access was measured as travel time in minutes to primary and secondary care facilities.

Several questionnaire variables were excluded from final analysis. We excluded variables that showed lack of variation between groups, contributed minimally to model fit, or had limited relevance to our primary research objectives. Upon review, some questionnaire items had design limitations or insufficient relevance to our analytical framework, precluding their inclusion in the primary analysis. A full descriptive table for all questionnaire variables is provided in Table S1.

For analytical purposes variables were classified as follows. Age and gender were included for demographic standardization and were therefore classified as adjustment variables. Socioeconomic status and maternal characteristics were classified as potential confounders. Nutritional diversity, supplement count, healthcare access, vaccination status, and birthweight were classified as mediators. Lastly, effect modifiers were comprised of gender and socioeconomic status, which were tested for interactions with altitude.

### 2.7 Measurements and tests

All members of the research team were trained in the technical procedures of the various measurements. The technical procedure protocols were strictly followed by the team members and were supervised by the primary investigator and supervisors.

The measurements were collected in the following order:

#### 2.7.1 Height and weight

Height was measured to the nearest 0.5 cm, with the child standing facing away from a stadiometer or tape measure attached to the wall of the examination room. Children were measured without shoes, assuming standard anatomical position, with the head in the Frankfurt position. Weight was measured with a digital or analog scale, to the nearest 0.05 kg, with the child lightly clothed and without shoes. The measuring tape and scale were provided by the participating schools. Calibration of the scales could not be verified prior to measurements, representing a potential source of measurement error.

#### 2.7.2 Waist circumference and mid‐upper arm circumference

WC was measured using a standard measuring tape to the nearest 0.5 cm. Measurements were taken at the midpoint between the iliac crest and the bottom of the 12th ribs, with the child standing upright and breathing normally.

MUAC was measured using a standard measuring tape, to the nearest 0.5 cm. Measurements were taken at the midpoint between the acromion and olecranon, with the arm hanging loose and relaxed. Measurements were taken from both the left and right arm, and the average was calculated.

#### 2.7.3 Oxygen saturation and pulse rate

Oxygen saturation and pulse rate were measured with the child seated, breathing normally. After 30 s of rest, the values of SpO_2_ (%) and pulse rate (bpm) were recorded. Measurements were taken using a Nellcor PM10N portable SpO_2_ patient monitoring system (Medtronic, Minneapolis, MN, USA).

#### 2.7.4 Hand grip strength

Hand grip strength (HGS) was measured using a Vetek Constant electronic hand dynamometer (Vetek, Väddö, Sweden), with the child seated, gripping the dynamometer with maximum force for at least 5 s. Each child performed the test 3 times for each hand, with a 15‐s rest period between attempts. The greatest absolute value from each hand was recorded and used for analysis.

#### 2.7.5 Lung function

FEV_1_, FVC, and FEV_1_/FVC were measured using a MicroMedical Microloop spirometer (MicroMedical/Jaeger, Rochester, Kent, UK). The device was calibrated and cleaned according to the manufacturer's specifications, and spirometry was performed according to the American Thoracic Society and European Respiratory Society (ATS/ERS) guidelines (Graham et al., 2019). After three technically acceptable blows were achieved from a maximum of 8, the highest FEV_1_ and FVC values were recorded, and FEV_1_/FVC was calculated. Predicted values and *z*‐scores were determined using the ERS Global Lung Function Initiative (GLI) lung function calculator (European Respiratory Society, 2025).

#### 2.7.6 Hemoglobin

Hemoglobin was measured with a Hemocue Hb 201+ (Hemocue, Ängelholm, Sweden) analyzer using a pinprick of capillary blood. Children were instructed to warm their hands, and a single finger was selected for sampling. The finger was wiped twice with an ethanol wipe before being left to dry. With a sterile lancet, a researcher drew blood and wiped away the first blood response. Blood was collected with a Hemocue 201 cuvette for analysis. Values were recorded in mmol/l and converted to g/l by dividing by 0.06206. The device was calibrated and cleaned according to the manufacturer's instructions.

### 2.8 Data analysis and statistics

Sample size calculations were performed assuming a 10% difference between groups for lung function, hemoglobin levels, height and weight, with 80% power and *α* = 0.05. The parameter that required the largest number of participants was chosen as the minimum requirement. This led to a minimum sample size of 100 participants per group.

Data was collected on paper case report forms and was transferred to a Redcap database for storage. Data in Redcap was verified twice by researchers to reduce human error in data transfer and entry. “Prefer not to answer” and “Do not remember” answers in questionnaires were coded as NA responses and were removed from analysis.

Statistical analysis was done in RStudio version 4.3.3 (Rstudio Team, 2025). Descriptive statistics were analyzed, and an overview is presented in Table 1. Student's *t*‐tests were calculated, measuring the average difference between highland and lowland children across all examinations, as seen in Table 2. Regarding FEV_1_, FVC, and FEV_1_/FVC, percent predicted and *z*‐scores were calculated using a Caucasian reference population from the GLI calculator (European Respiratory Society, 2025), as seen in Table 3. To account for the multiple comparisons made, and limit the amount of false positive results, the Benjamini‐Hochberg algorithm was used to apply a false discovery rate (FDR) correction to the resulting *p* values. This was done in RStudio using the *p*.adjust function to output FDR *p* values.

**TABLE 1:** Participant table.

Child characteristics	Bishkek		Naryn	
	*n =* 123		*n =* 143	
	Value	%	Value	%
Sex				
Male	61	49.6%	76	53.1%
Female	62	50.4%	67	46.9%
Age				
10	31	25.2%	31	21.7%
11	64	52%	59	41.2%
12	28	22.8%	53	37.1%
Age	11.5 (±0.7)		11.6 (±0.8)	

Family characteristics	Value	*n*/*N* ^a^	Value	*n*/*N* ^a^
Married parents	114 (92.7%)	143/143	136 (95.1%)	143/143
Monthly income category		106/123		140/143
Less than 10,000 KGS (<98 €)	10 (9.4%)		38 (27.1%)	
10,001–22,000 KGS (≈98–216 €)	18 (17%)		42 (30%)	
22,001–30,000 KGS (≈216–294 €)	15 (14.2%)		28 (20%)	
30,001–40,000 KGS (≈294–393 €)	19 (17.9%)		11 (7.9%)	
40,001–60,000 KGS (≈393–589 €)	25 (23.6%)		13 (9.3%)	
60,001–100,000 KGS (≈589–983 €)	16 (15.1%)		7 (5.0%)	
Above 100,000 KGS (>983 €)	3 (2.8%)		1 (0.7%)	
Number of children in family	3.7 (±1.2)	122/123	4.5 (±1.3)	143/143

Maternal and early life characteristics	Value	*n*/*N* ^a^	Value	*n*/*N* ^a^
Birth altitude	896.3 m (±396.2)	122/123	2129.6 m (±379.5)	143/143
Birth weight	3349 g (±518.8)	114/123	3102.6 g (±457.2)	136/143
Maternal age^b^	28.1 years (±5.7)	122/123	27.8 years (±6.1)	141/143
Maternal height	161 cm (±6.1)	121/123	159.5 cm (±7.1)	123/143
Maternal BMI	26.4 (±4.6)	121/123	26.8 (±4.8)	121/143
Primarily breast feeding the first 6 months	118 (95.9%)	123/123	134 (94.4%)	142/143

Healthcare access	Value	*n*/*N* ^a^	Value	*n*/*N* ^a^
Time to primary care	18.2 min (±15.4)	123/123	15.4 min (±8.3)	143/143
Time to secondary care	38.3 min (±21.0)	123/123	25.9 min (±21.7)	143/143
Received all recommended vaccines.	114 (92.7%)	123/123	117 (81.8%)	143/143

*Note*: *n*/*N* = number of participants with available data/total participants, values presented as mean ± SD for continuous variables and *n* (%) for categorical variables.

Abbreviation: KGS, Kyrgyz som.

^a^ NA either due to “prefer not to answer” or “do not remember”.

^b^ Maternal age at birth of the examined child.

**TABLE 2:** Child examinations—Differences between Bishkek and Naryn children.

Variable	Bishkek (*n* = 123)	Naryn (*n* = 143)	Difference [95% CI]	FDR^a^ adj. *p* value
	Mean ± SD	Mean ± SD		
Height cm	146.4 ± 7.0	143.7 ± 7.5	2.7 [1, 4.5]	0.006
Weight kg	38.7 ± 8.9	36 ± 6.7	2.7 [0.8, 4.6]	0.008
BMI	17.9 ± 3.0	17.3 ± 2.3	0.6 [−0.1, 1.2]	0.08
HAZ	−0.1 ± 1.0	−0.6 ± 0.9	0.5 [0.3, 0.7]	<0.001
BAZ	−0.0 ± 1.2	−0.3 ± 1.0	0.3 [−0.03, 0.5]	0.086
WC cm	63.9 ± 8.9	61.2 ± 6.05	2.7 [0.9, 4.6]	0.007
MUAC cm	21.2 ± 2.5	20.5 ± 2.1	0.7 [0.1, 1.2]	0.023
Hemoglobin g/L	120.2 ± 11.7	134.6 ± 13.6	−14.4 [−17.5, −11.4]	<0.001
Saturation %	97.6 ± 1.2	95.5 ± 1.6	2.1 [1.8, 2.5]	<0.001
HGS kgf	19.4 ± 3.6	20.7 ± 4.2	−1.3 [−2.2, −0.3]	0.013

^a^ The Benjamini‐Hochberg algorithm was used to generate FDR adjusted *p* values to limit the amount of false positive results.

**TABLE 3:** Child examinations—Lung function—Differences between Bishkek and Naryn children.

Variable	Bishkek (*n* = 103)	Naryn (*n* = 97)	Difference [95% CI]	FDR^a^ adj. *p* value
	Mean ± SD	Mean ± SD		
FEV_1_ L	2.2 ± 0.4	2.2 ± 0.4	0.0 L [−0.1, 0.1]	0.69
FVC L	2.5 ± 0.4	2.5 ± 0.4	0.0 L [−0.1, 0.1]	0.69
FEV_1_/FVC	86.3 ± 5.5	86 ± 4.9	0.3% [−1.2, 1.75]	0.69
FEV_1_ pp	93.7 ± 11.2	98.3 ± 10.6	−4.6% [−7.7, −1.6]	0.006
FVC pp	94.6 ± 11	99.8 ± 10	−5.2% [−8.2, −2.2]	0.003
FEV_1_/FVC pp	98.6 ± 6.1	98.1 ± 5.4	0.4% [−1.2, 2.1]	0.69
FEV_1_ z	−0.5 ± 1	−0.1 ± 0.9	−0.4 [−0.7, −0.1]	0.006
FVC z	−0.5 ± 0.95	−0.02 ± 0.9	−0.48 [−0.7, −0.2]	0.003
FEV_1_/FVC z	−0.1 ± 0.9	−0.2 ± 0.8	0.1 [−0.15, 0.3]	0.69

*Note*: 97/143 children in Naryn and 103/123 children in Bishkek fulfilled the BTS guidelines for good spirometry and were used for analysis. Percent predicted and *z*‐scores were calculated against a Caucasian reference population, using GLI equations (European Respiratory Society, 2025).

^a^ The Benjamini‐Hochberg algorithm was used to generate FDR adjusted *p* values to limit the amount of false positive results.

To test each measurement for significant gender differences, linear regression models were made for each parameter, adding an interaction term for gender and adjusting for age.

Following *t*‐tests and assessment of gender interactions, full regression models were built for exploratory analysis of the following primary outcomes: height, BMI, hemoglobin, FEV_1_, and FVC. Initially, all theoretically relevant variables were included: altitude location, demographics, income category, maternal factors, nutritional diversity, healthcare access, vaccination status, and birthweight. Subsequently, through backwards elimination, variables were removed in order of least significance, with a *p* value >0.10. While removing variables, the primary altitude exposure estimate and model fit were monitored, along with standard error, *R* squared and number of missing observations. The final models retained variables that were statistically significant, biologically important or whose removal substantially changed the altitude exposure estimate. When arriving at the final models, the remaining variables were tested for biologically plausible interaction terms. This resulted in five distinct linear regression models. Height, FEV_1_, and FVC can be found as Tables 4 and 5. BMI and hemoglobin can be found as Tables S2 and S3.

**TABLE 4:** Linear regression—Height in cm—Location gender interaction.

	Estimate [95% CI]	Std. error	t.value	*p* Value
Intercept	56.51 [33.71, 79.30]	11.56	4.89	<0.001
Location (ref: Lowland)	−0.20 [−2.48, 2.09]	1.16	−0.169	0.866
Gender (ref: Female)	−0.25 [−2.56, 2.05]	1.17	−0.217	0.829
Age (years)	4.78 [3.75, 5.81]	0.52	9.16	<0.001
Income category	0.44 [−0.05, 0.94]	0.25	1.76	0.080
Maternal height (cm)	0.13 [0.004, 0.25]	0.06	2.04	0.043
Maternal weight (kg)	0.09 [0.02, 0.16]	0.03	2.67	0.008
Birth weight (grams)	0.002 [0.0004, 0.004]	0.0008	2.43	0.016
Location gender interaction	−3.60 [−6.74, −0.48]	1.59	−2.27	0.024
Gender specific altitude effect				
Location—Boys	−3.80 [−6.19, −1.42]	1.21	−3.15	0.002
Location—Girls	−0.20 [−2.48, 2.09]	1.16	−0.17	0.866

*Note*: Complete data for all model variables was available for 215 of 266 participants (80.8%). Residual standard error: 5.697 on 206 degrees of freedom. Multiple *R*‐squared: 0.3995, Adjusted *R*‐squared: 0.3762 *F*‐statistic: 17.13 on 8 and 206 DF, *p* value: <2.2e‐16.

**TABLE 5:** Linear regression—Lung function FEV_1_ and FVC in liters.

	Estimate [95% CI]	Std. error	t.value	*p* Value
FEV_1_				
Intercept	−2.60 [−3.45, −1.75]	0.43	−6.02	<0.001
Location (ref: Lowland)	0.17 [0.095, 0.24]	0.04	4.594	<0.001
Gender (ref: Female)	0.09 [0.02, 0.16]	0.03	2.62	0.009
Height (cm)	0.03 [0.02, 0.03]	0.003	8.10	<0.001
Weight (kg)	0.01 [0.008, 0.019]	0.003	4.87	<0.001
Income category	0.03 [0.006, 0.05]	0.01	2.58	0.011
Vaccination count	0.02 [−0.002, 0.04]	0.01	1.76	0.080
FVC				
Intercept	−2.91 [−3.83, −1.99]	0.47	−6.25	<0.001
Location (ref: Lowland)	0.18 [0.10, 0.26]	0.04	4.57	<0.001
Gender (ref: Female)	0.19 [0.12, 0.26]	0.04	5.08	<0.001
Height (cm)	0.03 [0.02, 0.04]	0.004	8.51	<0.001
Weight (cm)	0.02 [0.01, 0.02]	0.003	5.91	<0.001
Income category	0.02 [−0.004, 0.04]	0.01	1.60	0.112
Vaccination count	0.02 [−0.009, 0.04]	0.01	1.223	0.223

*Note*: FEV_1_ Model: Complete data for all model variables was available for 182 of 200 participants (91%). Residual standard error: 0.2455 on 175 degrees of freedom. Multiple *R*‐squared: 0.6574, Adjusted *R*‐squared: 0.6457, *F*‐statistic: 55.97 on 6 and 175 DF, *p* value: <2.2e‐16. FVC Model: Complete data for all model variables was available for 182 of 200 participants (91%). Residual standard error: 0.2276 on 175 degrees of freedom. Multiple *R*‐squared: 0.6113, Adjusted *R*‐squared: 0.598 *F*‐statistic: 45.87 on 6 and 175 DF, *p* value: <2.2e‐16.

For further analysis, linear regression models were built for FEV1 and FVC with a categorical variable of the child's pneumococcal vaccine status (having received all, 2, 5, and 12 months, or having an incomplete vaccine regimen), while controlling for location, height, weight, gender, and income category. Pneumococcal regression for FEV1 and FVC can be found as Tables S4 and S5. Hand grip strength was explored separately using linear regression, controlling for gender, age, height, and weight. Hand grip strength regression can be found as Table S6.

To assess known associations between altitude and birthweight, we built a regression model testing birthweight and study location found as Table S7. To assess the robustness of our primary findings, in relation to an alternative exposure classification, sensitivity analysis was conducted using a binary threshold of ≥2000 MASL and <2000 MASL based on birth location. This alternative exposure was applied to the birthweight model, found as Table S8, as well as height, FEV1, and FVC, found as Table S9.

## 3 RESULTS

### 3.1 Participant characteristics

A total of 317 children were screened for eligibility, of whom 51 were excluded. Thus, 266 children were included in the final analysis: 123 from Bishkek and 143 from Naryn (Figure 1).

**FIGURE 1:**
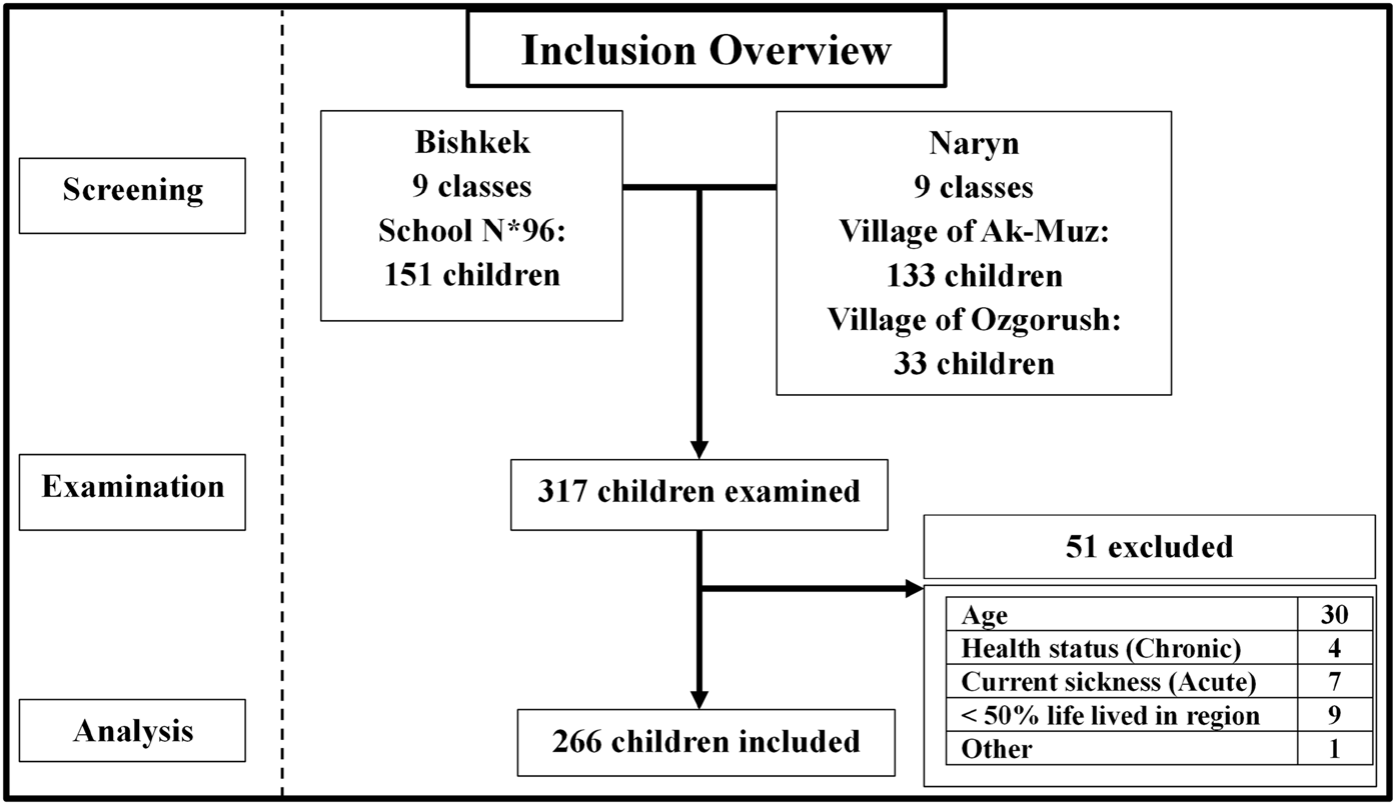
Inclusion table.

The study population was evenly distributed by sex, with a mean age of 11.6 in Naryn and 11.5 in Bishkek, as seen in Table 1. The family characteristics mainly differed regarding the number of children and monthly incomes. Household income was reported in Kyrgyz som (KGS), the official currency of Kyrgyzstan. The most common monthly income category for the Naryn families was 10.001–22.000 KGS, whereas the median income category was 22.001–30.000 KGS. In Bishkek, the most common monthly income category as well as the median monthly income category was 40.001–60.000 KGS. The maternal and early life characteristics are similar when considering maternal age, height, and BMI, and differ specifically in child birthweight and birth altitude.

### 3.2 Group comparisons

Table 2 shows Student's *t*‐test results, comparing children raised in Bishkek to Naryn. Children in Bishkek were significantly taller and significantly heavier in weight compared to their counterparts in Naryn. Their BMI and BAZ did not differ significantly, whereas their HAZ did differ significantly. Children in Bishkek had significantly larger WC and MUAC compared to the children in Naryn. Children growing up in Naryn had significantly stronger HGS, significantly higher hemoglobin levels, and significantly lower SpO_2_ levels.

Table 3 shows Student's *t*‐test results, between children raised in Bishkek and Naryn across FEV_1_, FVC, and FEV_1_/FVC, as well as percent predicted and *z*‐score values. Children in Bishkek and Naryn did not significantly differ in unadjusted FEV_1_, FVC, and FEV_1_/FVC. When using a Caucasian reference population, children in Naryn had significantly higher FEV_1_ percent predicted and FVC percent predicted compared to children from Bishkek. This significant pattern was also found in the following *z*‐scores. The FEV_1_/FVC ratio remained nonsignificant both concerning the percent predicted values and *z*‐scores.

### 3.3 Regression analysis

Figure 2 shows the results of several linear regression models, testing the location‐gender interaction across all measured parameters except lung function. Of all tested parameters, only height and HAZ were found to have significant gender‐location interactions. Male children growing up in Bishkek were significantly taller than their counterparts in Naryn, adjusted for age. Female children from Bishkek were not found to be significantly taller than their counterparts in Naryn. The location effect between genders was significantly different, meaning the altitude effect on height was significant for males, but not for females. This pattern was replicated in HAZ calculations.

**FIGURE 2:**
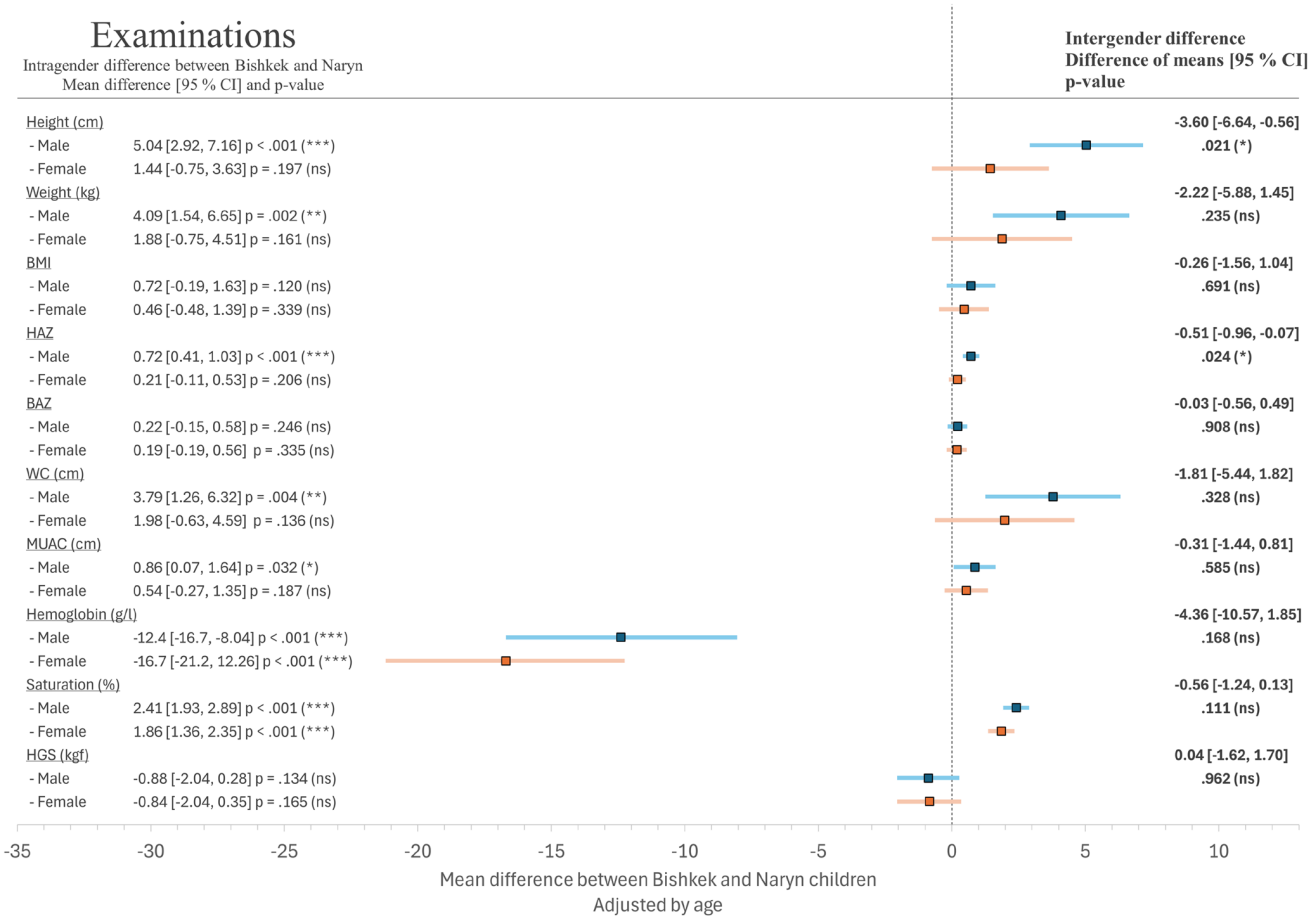
Differences in anthropometrics between Bishkek and Naryn children, stratified by gender. Forest plot displaying regression coefficient estimates for study parameters by location and gender. Left column: Mean differences with 95% confidence intervals comparing children from Bishkek versus Naryn (reference), stratified by gender. Right column: Gender‐location interaction effects with 95% confidence intervals, representing the intergender difference of altitude effect. Point estimates are shown as squares with horizontal lines indicating confidence intervals. Statistical significance levels are denoted as: *p < 0.05, **p < 0.01, ***p < 0.001.

Subsequent regression analysis showed that height continued to be significantly different between children from Naryn and Bishkek when controlling covariates such as demographics, income category, maternal factors, and birth weight. The interaction term between altitude and gender continued to be statistically significant. Though the effect estimate was slightly attenuated by the covariates, the difference remained robust and was not explained by the included covariates. Results of the full regression model can be seen in Table 4.

Table 5 shows linear regression models for FEV_1_ and FVC, controlled for appropriate covariates. FEV_1_ and FVC remained significantly different between Naryn and Bishkek children. With Naryn children having approximately 7.7% larger FEV1 and 7.2% larger FVC. In addition, Tables S4 and S5 show models for FEV_1_ and FVC adjusted for the same covariates, with the addition of pneumococcal vaccination status instead of overall vaccination count. FEV_1_ was significantly higher in children who were fully vaccinated against pneumococci. In contrast, FVC showed no significant difference.

Tables S2, S3, and S6 show that the difference in BMI became further diminished when controlling appropriate covariates, whereas hemoglobin and HGS remained significantly higher in children raised in Naryn.

Table S7 shows that birthweight was significantly lower among children in Naryn compared to Bishkek. Similarly, Table S8 shows that children born at an altitude equal to or greater than 2000 MASL were significantly lighter in birthweight compared to children born at less than 2000 MASL.

Sensitivity analysis in Table S9 shows that the alternative altitude threshold attenuated interaction effects that gender and location had on height, compared to the primary analysis found in Table 4. Although the direction and magnitude remained similar. FEV1 and FVC effect estimates remained similar and significant throughout the sensitivity analysis.

## 4 DISCUSSION

Following independence in 1991, Kyrgyzstan implemented numerous policies to restructure and strengthen its food security and health system in general, but especially also aiming to assist impoverished rural families. Among others this was done by creating an economy on small household farms and adopting global health protocols, working with UNICEF and WHO (Akseer et al., 2018). Following these implementations, Kyrgyzstan substantially reduced childhood malnutrition, shown through several parameters but especially by way of stunting. Monitoring of childhood nutrition and development continues through collaboration with UNICEF and the national DHS and MICS surveys. While the continued monitoring of childhood malnutrition is essential in tracking the impact of healthcare initiatives, it is not very sensitive to specific regional differences in the developmental environment (Leroy & Frongillo, 2019; Scheffler et al., 2020). Therefore, researching how children develop differently between regions could offer unique insights for policy makers and healthcare professionals.

We found that children raised in highland Naryn were significantly shorter and lighter but had superior lung function and higher hemoglobin levels compared to lowland Bishkek children. The changes in height and weight may be due to poor nutrition, lower socioeconomic status, and poorer access to healthcare, determinants which are often associated with the exact rural communities at high‐altitude (Greksa, 2006; Shively & Schmiess, 2021) which the Kyrgyz government has tried to support. While the children from Naryn did come from a rural environment with a lower median income category and lower vaccination completion, our outcomes did not change when controlling for these covariates. Therefore, we hypothesize that these parameters should not be examined in isolation, as the children also had superior lung function, stronger hand grip strength, and higher hemoglobin levels. Adaptations which are either associated with healthy development and strength or residing at high‐altitude and not necessarily associated with malnourished populations (Al‐Hashem, 2006; Bustamante et al., 2022; Frisancho, 2013; Greksa, 2006; Greksa et al., 1988). Consequently, the lower stature and weight may instead be due to development at high‐altitude under hypobaric hypoxia (Baye & Hirvonen, 2020; Ortiz‐Prado, Mendieta, et al., 2022; Shively & Schmiess, 2021). Authors have previously suggested that this may be due to an adaptive response in the fetus that maximizes oxygen delivery through a smaller placenta. Subsequently, during child growth, the body directs available energy to the critical oxygen transporting organs rather than the musculoskeletal system (Greksa, 2006).

We found support for this hypothesis in our analysis, where birthweight was significantly lower among children born at higher altitude, both when comparing Bishkek to Naryn and when applying our alternative altitude threshold, consistent with documented associations between altitude exposure and reduced fetal growth (Suri et al., 2025). Nevertheless, birthweight only explained part of the observed differences in child height. Even after adjusting for birthweight, altitude remained significantly associated with reduced height, an effect specifically found in male children. While this suggests that in‐utero hypoxic exposure may contribute to the growth differences observed, mechanisms operating during postnatal development may also play an independent role.

Within these altitude related growth patterns, and independent of the birthweight related mechanism described above, we observed a notable difference between genders. Interestingly, male children seem to be affected more, in linear growth, than female children. This difference could be due to slowed pubertal growth caused by altitude (Sanchez‐Macedo et al., 2024), but it is difficult to say if this gap would persist into adulthood. It has been shown that age and height of onset of the pubertal growth spurt (PGS) are highly correlated with final height in normal puberty (Limony et al., 2015). A similar pattern was reported in adults by Ortiz‐Prado, Mendieta, et al. (2022) where their study from 2022 found that adult men living in the Andean highlands were shorter than their low‐altitude counterparts and had significantly more muscle body mass. Whereas women had less muscle body mass but were not significantly different in height. Although their study corroborates our findings in male children, several others have found both genders at high altitude were significantly impacted in linear growth, without an intergender difference (Ortiz‐Prado, Encalada, et al., 2022; Ulrich et al., 2019). Adding to this, our study did not control puberty status of the examined children, and therefore the gender difference could in fact simply be due to a mismatch in pubertal status, and not a physiological trait caused by high‐altitude. Additionally, the non‐significant interaction observed under the alternative birth‐altitude threshold in Table S9 may partly reflect that this classification captures only birth location, whereas our primary exposure reflects the location where children resided for most of their lives. Children born at one altitude who subsequently moved to and predominantly resided at the other may have been misclassified under this stricter threshold, potentially diluting the observed interaction effect.

Beyond growth parameters, when focusing on hematological and respiratory measures of adaptation and acclimatization, the children of Naryn did have significantly higher levels of hemoglobin, lower SpO_2_, as well as higher FEV_1_ and FVC values. This pattern of development is well known from earlier studies (Al‐Hashem, 2006; Bustamante et al., 2022; Frisancho, 2013; Greksa, 2006; Greksa et al., 1988), but especially increased lung function has recently been found in healthy non‐smoking Kyrgyz adults living at high altitude, by Ulrich et al. (2019). In their study, the adult low‐altitude and high‐altitude samples were from roughly the same region as our child samples. Unlike height, birthweight did not reach the significance for retention in our final FEV1 and FVC models during backward elimination. This likely reflects that its association with lung function was mostly accounted for by current height. This could suggest that the respiratory findings are less likely to be dependent on birthweight in‐utero related exposure, compared to linear growth. This could instead reflect postnatal physiological adaptation to chronic hypoxia.

In contrast to these physiological adaptations, other anthropometric measures showed more modest differences. While the children of Bishkek had slightly larger WC and MUAC, the implications of these differences might be negligible, as the differences in BMI and BAZ were nonsignificant. If anything, they could be an indication of increased access to more calorie dense nutrition in Bishkek, compared to the rest of the country (National Statistical Committee of the Kyrgyz Republic & UNICEF, 2024), leading to near identical BMI's but different body compositions of the children. Subsequently, children in Naryn had stronger hand grip strength, which has been associated with total muscle strength in both pediatric patients and healthy children (McKirdy et al., 2021; Wind et al., 2010). While this could lead to the belief that children in Naryn were more physically fit than their counterparts in Bishkek, it is important to note that we have not directly measured their fitness or activity levels. In this sense, hand grip strength simply serves as a proxy that may or may not be associated with total muscle strength.

Further analysis of the regression models for lung function indicated that completion of the full pneumococcal vaccination schedule was associated with higher FEV1 values, independent of altitude, gender, height, weight, and household income. While this observation is consistent with suggested respiratory protective effects of pneumococcal vaccination (Collaro et al., 2023; Dharmage et al., 2009), it should be interpreted with caution due to the very small number of unvaccinated children in our sample (*n* = 5 in Bishkek, *n* = 6 in Naryn) and the inability to infer causality.

The broad approach of this study offers several strengths towards the results. Testing several parameters allows for increased granularity in assessing growth of children and opens room for a nuanced discussion of their development. Although our study performed several direct comparisons, increasing the risk of false positives, controlling through FDR adjusted p‐values still left all mentioned results at statistical significance, further strengthening the found associations. A detailed questionnaire for the parents of the examined children allowed for control of demographics, environmental, and genetic covariates. The sampled populations had comparable distributions of age, gender, and outliers, and were therefore suitable for structured comparisons. Furthermore, we gathered information about birth location and length of stay at altitude, further strengthening the comparability of our samples. To be included in the study, the children had to live at least 50% of their lifetime at the study location; this was stated as an inclusion criterion to limit migration noise in our samples. Some may point out that a 50% limit is too little exposure when studying high altitude, but we see it as a strength, as this study isn't inherently interested in in‐utero altitude exposure, compared to childhood development after birth. While national surveys such as DHS and MICS focus on children up to 5 years of age and their mothers, our study brings to light how some of these children develop towards adolescence, strengthening the information on childhood development in Kyrgyzstan. Additionally, the sampling of children from Naryn and Bishkek is roughly the same as previous lung function research done in adults in Kyrgyzstan, further strengthening comparability of our findings. Lastly, the collaborative work between researchers from Denmark and Kyrgyzstan during this study strengthens global efforts to engage health workers in combined research and innovation.

Our study may have several limitations influencing the results. Firstly, we must acknowledge a certain amount of differential selection bias. In the Naryn region, participants seemed to be largely driven by healthcare scarcity, due to less frequent access to healthcare. This led to positive pressure for participation, arguably leading to a representative sample of the highland population. In contrast, citizens of the urban capital might have more confidence in their child's healthy development due to easier access to healthcare and a higher socioeconomic status. This may either cause participants to only engage if they have specific healthcare concerns or have an explicit interest in healthy development, both representing self‐selection away from the average lowland population. This weakened the representativeness of the Bishkek sample and the external validity of our comparison. This limitation is difficult to avoid in studies comparing populations with structurally unequal access to healthcare. Secondly, while choosing an age group, a balance between altitude exposure duration, quality of examinations and limited participants in puberty was attempted, but we acknowledge that some of the children in the group 10–12 years were probably already in puberty, especially the female children. While an examination and subsequent control of puberty status would have been ideal, this was not feasible in our study design. Despite attempting to ensure strict measurement protocols, no tests were done to assess observer variability or to ensure repeatability and reproducibility in measurements (Popović & Thomas, 2017). While others emphasize controlling ethnicity, our study focused on inhabitants of the examined locations and not on their genetic or phenotypic ethnicity (Facchini et al., 2007; Ortiz‐Prado, Encalada, et al., 2022; Ulrich et al., 2019). This may hinder the data's value as reference material but does not preclude hypothesis generation about the Kyrgyz population.

Kyrgyzstan struggles with air quality, particularly in Bishkek during winter months (United Nations Development Programme & United Nations Environment Programme, 2022). We cannot exclude differences in chronic air pollution exposure between the high‐altitude rural and low‐altitude urban settings. Urban air pollution in Bishkek is largely driven by traffic emissions and coal‐based heating during winter, whereas rural high‐altitude areas rely predominantly on household solid‐fuel heating, which may contribute to indoor air pollution. These potential differences were not directly assessed and should be considered when interpreting respiratory outcomes. While research has recently been done to create a respiratory reference population for healthy Kyrgyz adults (Ulrich et al., 2019), none exists for children yet. Therefore, we used a reference population of Caucasian origin, which could skew results. While measurements at the same time of day for all children would have been ideal for both the lung function, hemoglobin and HGS measurements, this was simply not economically or logistically feasible for our research team. Lastly it is important to note that the children in the highlands of Naryn were examined during late winter and the children in Bishkek were examined during early summer, creating a stark contrast in environment when examined.

While our findings are statistically significant, their clinical significance remains to be discussed. One of our main findings states that children raised at high altitude are 2.7 cm or 0.5 SD shorter than their lowland counterparts. Following further analysis, we found that the difference in height was primarily driven by the male children, and this difference might be due to a lack of puberty control in our study design. Therefore, the discussion of linear growth in this sample should be considered with caution, until our findings can be evaluated in future research. Taking this into consideration, 2.7 cm in linear growth might not be clinically meaningful when observing individuals; however, a difference of 0.5 SD seen on a population basis is clinically meaningful. If their environment predisposes them to be 0.5 SD closer to the stunting definition, then it should be considered if these children should be assessed in other ways than through linear growth alone.

Lung function and hemoglobin are classic parameters that have previously been shown to be affected in altitude research (Al‐Hashem, 2006; Bustamante et al., 2022; Frisancho, 2013; Greksa, 2006; Greksa et al., 1988). Our child samples were shown to have a rough difference of 7% in FEV_1_ and FVC, and a 14 g/L difference in hemoglobin levels. Both clinically significant differences when assessing a patient, as it would be important to consider that these children develop in an environment that has an increased demand in nutrition and respiratory capacity.

Lastly, the children had a difference in HGS of 1.3 kg. A 1‐kg difference corresponds to the average developmental increase seen over an entire year in Dutch reference populations (Wind et al., 2010), suggesting potential clinical relevance for muscle strength assessment, though its impact on continued development requires further investigation.

While much research on high‐altitude adaptation and acclimatization has been done in Andean and Tibetan populations (Tremblay, 2024), inferring these results directly to high‐altitude residents in Kyrgyzstan is difficult. In part due to the unique make‐up of the Kyrgyz population, mainly influenced by the vast migration across several time periods, including when the silk road was most active (Comas et al., 1998). While some research has been done during the USSR, internationally published high‐altitude research in Kyrgyzstan has only recently been intensified by local and international researchers. Taking this into consideration, Kyrgyzstan is uniquely poised for continued research involving the health of children at high‐altitude regions. Therefore, future research could employ longitudinal study designs, tracking children into adolescence and adulthood to determine whether the observed gender differences in height persist, and whether highland children's functional advantages translate into long‐term health benefits or limitations. To expand on causal inference regarding the altitude effects, comparative studies across multiple altitude gradients within Kyrgyzstan would be needed. Lastly, more targeted intervention studies regarding specific nutritional supplementation could provide evidence‐based guidance for pediatric care in high‐altitude settings.

## 5 CONCLUSION

In summary, our study demonstrates that healthy Kyrgyzstani schoolchildren raised at high altitude exhibit a distinct developmental pattern characterized by reduced linear growth but superior respiratory function, hematological levels, and muscular hand grip strength. While birthweight was lower among children born at higher altitude and partially explained the observed reduction in height, it did not account for the gender specific growth pattern or our respiratory findings, suggesting a broader adaptive response to hypobaric hypoxia, extending beyond in‐utero exposure alone, rather than malnutrition as the sole explanation.

Our findings highlight the need to interpret WHO growth standards with caution when considering high altitude pediatric populations. While these findings may show a developmental profile supported by adaptation and acclimatization rather than malnutrition, our findings are merely observational. Albeit these associations may suggest that investments in child health in Kyrgyzstan are succeeding, this does not by any means call for less investment in pediatric healthcare. If anything, it implies that attention to childcare and development should be of continued focus. This is exactly because the development of these children is influenced by multiple factors and is hard to interpret by traditional standards, such as linear growth and standard reference material.

Longitudinal studies tracking the development of children at varying altitudes across Kyrgyzstan are needed to disentangle the long‐term effects of altitude‐dependent environmental exposures and socio‐economic determinants. These investigations would hold significant value for both national health planning and the continued international scientific understanding of pediatric high‐altitude physiology.

## AUTHOR CONTRIBUTIONS

**Adam Bøcker Toft Olsen:** Conceptualization; data curation; formal analysis; investigation; methodology; project administration; resources; software; visualization. **Elvira Isaeva:** Conceptualization; data curation; formal analysis; investigation; methodology; project administration; resources; supervision; validation. **Madina Umetalieva:** Data curation; investigation; resources; validation. **Nargiza Osmonbaeva:** Data curation; investigation; resources; validation. **Sofie Valborg Wright:** Data curation; investigation; validation. **Lea Deniz Lund Serefoglu:** Data curation; investigation; resources; validation. **Freya Natasha Kretzchmar:** Data curation; investigation; validation. **Trisse Dyrløv Høegh:** Data curation; investigation; validation. **Jesper Kjærgaard:** Conceptualization; methodology; resources. **Kim Gjerum Nielsen:** Investigation; methodology; resources. **Talant Sooronbaev:** Conceptualization; funding acquisition; project administration; supervision. **Anja Poulsen:** Conceptualization; funding acquisition; methodology; project administration; resources; supervision.

## FUNDING INFORMATION

This research was funded by GLOBUS—the fund for global engagement, education and collaboration of the Ministry of Foreign Affairs of Denmark.

## CONFLICT OF INTEREST STATEMENT

On behalf of all authors, the corresponding author states that there is no conflict of interest. No financial or non‐financial benefits have been received or will be received from any party related directly or indirectly to the subject of this article.

## ETHICS STATEMENT

Ethical approval by the Ethics Committee for Conducting Clinical Trials and Biomedical Research at the National center of Maternity and Childhood Care of the Ministry of Health and Social Development of the Kyrgyz Republic. February 7, 2025.

## USE OF ARTIFICIAL INTELLIGENCE

Olsen, A. B. T. used Claude Sonnet 4.5–4.6 (Anthropic, San Francisco CA, USA) for assistance in R code generation and debugging. In addition, Claude 4.6 Sonnet was utilized for general text clarity and grammatical proofreading. All code generated by AI was critically inspected and reviewed for errors; likewise, all sections revised or edited by AI were inspected by the corresponding author. Authors take full responsibility for the content contained in the manuscript and all data generated by the corresponding code. No patient information or generated data was shared with AI models, and all data contained and analyzed was anonymized before being managed.

## Supporting information


Data S1.

## Data Availability

In accordance with the informed consent form signed by all participating parents, all information collected must be kept confidential and only the research team may have direct access to the data. Public access to the datasets generated and/or analyzed during the current study is therefore restricted. Access to the anonymized dataset is only available from the corresponding author upon reasonable request.
