# Supplementary material for: Altitude and child development: A cross‐sectional comparative study of children in urbanized low‐altitude and rural high‐altitude settings in Kyrgyzstan
Source: Physiol Rep. 2026 Oct 12;14(19):e71132. doi: 10.14814/phy2.71132 (PMC13658936; doi:10.14814/phy2.71132)
Supplement: Supplementary file 1 [file PHY2-14-e71132-s001.zip › PHYSREP-2026-04-377-T-s04.pdf]

Только для ассистента исследователя: *For research assistant only:*

ID участника: ______________ *Дата:* _______________
Participant ID: *Date:*

*ID исследователя (RA ID)*____________________________

Анкета родителя/опекуна

Questionnaire – Parent / Guardian

Эта анкета предназначена для родителя данного ребенка или его законного опекуна.
This questionnaire is meant for the mother of the child being examined, or the guardian.

ФИО родителя/опекуна: Parent / Guardian full name ____

ФИО ребенка: Child's full name ____

Контактную информацию можно найти в форме согласия.
Contact information can be found on the consent form.

Пожалуйста, заполните следующую анкету, поставьте «✓» в соответствующем ответе. Если вы хотите оставить комментарии, запишите их в «Примечания». Если вы не хотите отвечать на какой-либо вопрос, отметьте «✓» в поле «Предпочитаю не отвечать» Please fill out the following questionnaire as best possible, with only an ✓ in the corresponding answer. If you need to expand on a topic, please do so in the corresponding "Notes" field. If you do not wish to answer a certain question, please mark it with an ✓ in the corresponding "prefer not to answer" field.

Если у вас есть вопросы относительно исследования, пожалуйста, не стесняйтесь обращаться к исследователям. Их контактную информацию можно найти в форме согласия.
If you have any inquiries regarding the study, please feel free to contact the researchers. Their contact information can be found on the consent form.

01. Семейное положение родителей данного ребенка Бул баланын ата-энесинин үй-бүлөлүк абалы

Marital status – Parents of the examined child

	**Да**/Yes	Нет/No
**Женаты/замужем**(Married)		
**Не женат/не замужем** (Single)		
**Разведены** (Divorced)		
**Вдова / Вдовец** (Widow / Widower)		
**Другое (уточните)** Other (specify)		
**Предпочитаю не отвечать:** Prefer not to answer:		
**Примечания:** Notes:		

02. Кто, кроме Вас проживает в доме? Сизден башка уйдо ким жашайт?

Who lives in the household, besides yourself?

	**Да**/Yes	**Нет**/No
**Супруг(а)** Spouse		
**Бабушка/дедушка** (Grandparents)		
**Родственники** (Relatives)		
**Живу один/одна** None (alone)		
**Другое (уточнить)** Others (specify)		
**Предпочитаю не отвечать:** Prefer not to answer		

Только для ассистента исследователя: *For research assistant only:*

ID участника: ____________ *Дата:*________________
Participant ID: *Date:*

ID исследователя *(RA ID)*___________________________

**Примечания:**<br>Notes:

03. Сколько у вас детей?/ Канча балаңыз бар?

How many children do you have?

**Сколько:**<br>How many:	
**Предпочитаю не отвечать:**<br>Prefer not to answer:	
**Примечания:**<br>Notes:	

04. Где родился Ваш ребенок?/Балаңыз кайда төрөлгөн?

Where was your child born??

**Местность (село/город), <u>подчеркнуть подходящее:</u>**<br>Locality (village/city*),* <u>line the correct answer:</u>	
**Указать высоту местности (только для АИ):**<br>Altitude of location (For RA only):	
**Предпочитаю не отвечать:**<br>Prefer not to answer:	

05. Возраст биологической матери данного ребенка? Баланын апасынын жашы канчада?

What is the age of the biological mother of this child?

**Возраст:**<br>The age	
**Предпочитаю не отвечать:**<br>Prefer not to answer:	

06. Какой примерно рост и вес <u>матери</u> данного ребенка? Баланын апасынын бою жана салмагы

What is the approximate height and weight of the <u>biological mother</u>, of the examined child?

**Рост, пожалуйста, укажите в сантиметрах (см).**<br>Height, please fill out in centimeters (not an x)	
**Вес, пожалуйста, укажите в килограммах (кг).**<br>Weight, please fill out in kilograms (not an x)	
**Предпочитаю не отвечать:**<br>Prefer not to answer:	

Только для ассистента исследователя: *For research assistant only:*

ID участника: ______________ *Дата:*_________________
Participant ID: *Date:*

*ID исследователя (RA ID)*_____________________________

07. Прикладывали ли ребенка к груди сразу после рождения? Төрөлгөндөн кийин балага эмчек эмиздиңиз беле?

Immediately after birth was the examined child put directly on the bare skin of the mothers chest?

**Да/**Yes	**Нет/**No
**Предпочитаю не отвечать:**<br>Prefer not to answer:	

08. Какой примерно был вес вашего ребенка при рождении? Баланын төрөлгөндөгу салмагы

What was the approximate weight of the examined child at birth?

**Вес, пожалуйста, укажите в килограммах (кг).**<br>Weight, please fill out in kilograms (not an x)	
**Не помню:** Don't remember:	
**Предпочитаю не отвечать:**<br>Prefer not to answer:	

09. Кормили ли вы вашего ребенка преимущественно грудным молоком до 6-ти месяцев? /6 айга чейин бала эмчек эмди беле?

Did you predominantly breastfeed the examined child, when the child was less than 6 months old?

**Да/**Yes	**Нет/**No
**Не помню:** Don't remember:	
**Предпочитаю не отвечать:**<br>Prefer not to answer:	

10. Давали ли вы вашему ребенку какие-либо из перечисленных ниже БАДы (биологически активные добавки)? Азыркы убакытка чейин балага кандайдыр бир БАК(биологикалык активдуу кошумчалар) берилди беле?

When raising the examined child, did you make use of any of the following dietary supplements?

	**Да/**Yes	**Нет/**No
**Цинк** Zinc		
**Витамин А** Vitamin A		
**Витамин Д** Vitamin D		
**Мультивитамины** Multivitamin		
**Гулазык** Gulazyk		
**Омега 3** Omega 3		
**Другое (уточнить)**<br>Other (specify in notes)		
**Предпочитаю не отвечать:**<br>Prefer not to answer:		

Только для ассистента исследователя: *For research assistant only:*

ID участника: ______________ *Дата:* ________________
Participant ID: *Date:*

ID исследователя (RA ID) ____________________________

**Примечания:**<br>Notes:

11. Используете ли вы йодированную соль в пище? Үйдо йодтолгон туз колдоносуңарбы?

Do you use iodized salt in your cooking?

**Да/**Yes	**Нет/**No
**Предпочитаю не отвечать:**<br>Prefer not to answer:	

12. Пил ли ваш ребенок какие-либо из перечисленных ниже жидкостей в течение последних 24 часов? 24 саattтын ичинде баланыз кандай суусундук ичкен?

Did your child drink any of the following liquids during the last 24 hours?

**Подчеркните тип жидкости в каждой категории «да».**<br>Please underline the type of liquid ingested, in each “yes” category.		
	**Да/**Yes	**Нет/**No
**Обычная вода**<br>Plain water		
**Фруктовый сок, компот или напитки с фруктовым вкусом. В том числе напитки, приготовленные из сиропов или порошков.**<br>Fruit juice, kompot or fruit flavoured drinks. Including drinks made from syrups or powders.		
**Шорпо, бульон.**<br>Shorpo, clear broth.		
**Молоко животного происхождения**<br>Milk from animals.		
**Йогурт, кефир, айран, биолакт, кымыз**<br>Yogurt drinks, such as kefir, ayran, biolact or kymyz.		
**Шоколадные напитки, включая из сиропов или порошков? Какао?**<br>Chocolate flavoured drinks including those made from syrups or powders? Cocoa?		
**Мальтные напитки (жарма, максым, бозо)**<br>Malt drinks (jarma, maksym, bozo)?		
**Газированные напитки (Кока-Кола, Фанта, Спрайт, Пепси, лимонад и т.д.)**<br>Soda (Coca-Cola, Fanta, Sprite, Pepsi, lemonade, etc.)		
**Энергетические напитки (Нитро, Флэш, Горилла и т.д.)**<br>Sport drinks or energy drinks (Nitro, Flash, Gorilla etc.)		
**Чай, айс-ти, кофе или травяные напитки?**<br>Tea, Ice tea, coffee or herbal drinks?		
**Другое (уточните)** Other (specify)		
**Предпочитаю не отвечать:**<br>Prefer not to answer:		
**Примечания:**<br>Notes:		

Только для ассистента исследователя: *For research assistant only:*

ID участника: ____________ *Дата:* ______________
Participant ID: *Date:*

*ID исследователя (RA ID)*________________________

13. Ел ли ваш ребенок какие-либо из следующих продуктов в течение последних 24 часов? Балаңыз акыркы 24 сааттын ичинде төмөнкү тамактардын бирин жедиби?

Did the examined child eat any of the following food groups during the last 24 hours?

**Подчеркните тип потребляемой пищи в каждой категории «да».** Please underline the type of food ingested, in each "yes" category.		
	**Да/**Yes	**Нет/**No
**Йогурт** Yogurt		
**Каша, хлеб, рис, макароны, вермишель, паста или другие продукты, изготовленные из злаков** Porridge, bread, rice, noodles, bulamyc, vermicelli, pasta, or other foods made from grains		
**Тыква, морковь, кабачки или сладкий красный перец.** Pumpkin, carrots, squash, or sweet red peppers		
**Картофель или репка** Potatoes or turnips		
**Темно-зеленые листовые овощи, такие как китайская капуста, шпинат, брокколи, руккола или щавель** Dark green, leafy vegetables, such as Chinese cabbage, spinach, broccoli, arugula or sorrel		
**Любые другие овощи, такие как помидоры, огурцы, баклажаны, зеленый перец, цветная капуста, редис, редька или свекла** Any other vegetables, such as tomatoes, cucumbers, eggplants, green peppers, cauliflower, radish, garden radish or beetroot		
**Абрикосы, сухофрукты из абрикосов, спелая хурма или дыня с оранжевой мякотью** Apricots, dried apricots, ripe persimmon or melon that is orange inside		
**Любые другие фрукты, такие как яблоки, груши, персики, бананы, виноград, малина, клубника, арбуз или апельсины** Any other fruits such as apples, pears, peaches, bananas, grapes, raspberry, strawberry, watermelon or oranges		
**Печень, почки, сердце или другие субпродукты** Liver, kidney, heart or other organ meats		
**Колбасы, хот-доги или консервированное мясо** Sausages, hot dogs, or canned meat		
**Любое другое мясо, такое как говядина, баранина, козлятина, конина, курица, утка или гусь** Any other meat, such as beef, lamb, goat, horse, chicken, duck or goose		
**Яйца** Eggs		
**Рыба, свежая или сушеная, или морепродукты** Fish, either fresh or dried, or shellfish		
**Бобы, горох, нут, чечевица, семена или орехи, такие как грецкие орехи, лесные орехи, миндаль или арахис** Beans, peas, chickpeas, lentils, seeds, or nuts, such as walnuts, hazelnuts, almonds or peanuts		
**Твердые или мягкие сыры, такие как пармезан, голландский сыр, тильзит, сулугуни, фета, моцарелла, творог или курут** Hard or soft cheese such as parmesan, Dutch cheese, tilsit, suluguni, feta, mozzarella. cottage cheese or qurut		
**Сметана, масло и другие молочные продукты** Sour cream, butter and other dairy products?		
**Сладкие продукты, такие как шоколад, конфеты, выпечка, торты, печенье или мороженое** Sweet foods such as chocolates, candies, pastries, cakes, biscuits or ice cream?		
**Чипсы, картофель фри, пельмени, самса, пирожки или беляши** Chips, crisps, puffs, French fries, boorsok, instant noodles, pies, samsa, pirozhki or belyashi?		
**Предпочитаю не отвечать:** Prefer not to answer:		

Только для ассистента исследователя: *For research assistant only:*

ID участника: ______________ *Дата:* _________________
Participant ID: *Date:*

*ID исследователя (RA ID)*_____________________________

**Другие (уточните):**<br>Other (specify):

14. Получил ли ваш ребенок все рекомендованные прививки согласно календарю прививок? Балаңыз бардык сунушталган эмдөөлөрдү эмдөө календарына ылайык алдыбы?

Did the child receive all recommended vaccinations, according to schedule?

**Какие вакцины получил ваш ребенок?**<br>Which vaccines did your child receive?	
**В роддоме** In the maternity hospital	
**2 месяца** 2 months	
**3,5 месяца** 3,5 months	
**5 месяцев** 5 months	
**9 месяцев** 9 months	
**12 месяцев** 12 months	
**2 года** 2 years	
**6 лет** 6 years	
**Предпочитаю не отвечать:**<br>Prefer not to answer:	
**Другое, уточните:**<br>Other, specify:	

15. Сколько времени вам понадобится, чтобы добраться до ближайшего медицинского учреждения? Жакынкы медициналык мекемеге жетүү үчүн канча убакыт талап кылынат?

How much time does it take for you to access your nearest primary care, and secondary care facility?

**До ЦСМ, ФАП (минуты/часы)**<br>Primary care (Time in minutes/hours)		**До больницы (минуты/часы)**<br>Secondary care (Time in minutes/hours)	
**Какой вид транспорта? отметьте «✓» в поле**<br>What type of transportation? Mark with a ✓		**Какой вид транспорта? отметьте «✓» в поле**<br>What type of transportation? Mark with a ✓	
**Пешком**<br>By foot		**Пешком**<br>By foot	
**На машине**<br>Car		**На машине**<br>Car	
**Другое**<br>Other		**Другое**<br>Other	
**Предпочитаю не отвечать:**<br>Prefer not to answer:			
**Примечания:**<br>Notes:			

Только для ассистента исследователя: For research assistant only:

ID участника: ______________ Дата: __________________
Participant ID: Date:

ID исследователя (RA ID)______________________________

16. Есть ли у вас электричество?/ Үйдө электр жарыгы барбы?

Do you have electricity?

	**Да/**Yes	**Нет/**No
**Централизованная сеть**<br>Centralized grid		
**Генератор/изолированная система**<br>Off-grid (generator/isolated system)		
**Нет электричества**<br>We do not have electricity		
**Предпочитаю не отвечать:**<br>Prefer not to answer:		
**Другое (укажите):**<br>Other (specify):		

17. Сколько вы зарабатываете в месяц (семейный бюджет)? Айына канча табасыз (үй-бүлөлүк бюджет)?

What do you earn in a month (family budget)?

	**Да/**Yes	**Нет/**No
**Менее 10.000 сом**<br>Less than 10.000 som		
**10.001-22.000 сом**<br>10.001-22.000 som		
**22.001-30.000 сом**<br>22.001-30.000 som		
**30.001-40.000 сом**<br>30.001-40.000 som		
**40.001-60.000 сом**<br>40.001-60.000 som		
**60.001-100.000 сом**<br>60.001-100.000 som		
**Более 100.000 сом**<br>Above 100.000 som		
**Предпочитаю не отвечать:**<br>Prefer not to answer:		
**Другое (укажите сумму):**<br>Other (amount):		

Только для ассистента исследователя: *For research assistant only:*

ID участника: ______________ *Дата:*_________________
Participant ID: *Date:*

*ID исследователя (RA ID)*_____________________________

18. Являетесь ли вы или кто-то из членов вашей семьи владельцем дома, в котором вы живете?
Сиз же сиздин үй-бүлө мүчөлөрүңүз сиз жашаган үйдүн ээсиби?

Do you or anyone else in your family own the house you live in?

	**Да/**Yes	**Нет/**No
**Собственный дом**<br>Own		
**Аренда**<br>Rent		
**Предпочитаю не отвечать**<br>Prefer not to answer		
**Другое (укажите):**<br>Other (specify):		

19. Где находится ваш источник воды (для питья)?
Сиздин суу булагыңыз кайда (ичүү үчүн)?

Where is your water source located (for drinking)?

	**Да/**Yes	**Нет/**No
**В собственном доме/квартире**<br>In own household/appartement		
**В собственном дворе**<br>In own yard		
**Предпочитают не отвечать**<br>Prefer not to answer		
**В другом месте (укажите):**<br>Elsewhere (specify):		

20. Курит ли кто-нибудь в семье?
Үй-бүлөдө бирөө тамеки тартабы?

Does anyone smoke in the household?

	**Отметьте ✓**<br>Mark with an ✓
**Никто** No one	
**Да, только вне дома**<br>Yes, outdoors only	
**Да, в помещении**<br>Yes, indoors	
**Курил раньше (сейчас бросил)**<br>Previously smoked (now quit)	
**Предпочитаю не отвечать** :<br>Prefer not to answer:	
**Другое, укажите в примечаниях:**<br>Other, specify in notes:	
