# Supplementary material for: Altitude and child development: A cross‐sectional comparative study of children in urbanized low‐altitude and rural high‐altitude settings in Kyrgyzstan
Source: Physiol Rep. 2026 Oct 12;14(19):e71132. doi: 10.14814/phy2.71132 (PMC13658936; doi:10.14814/phy2.71132)
Supplement: Supplementary file 1 [file PHY2-14-e71132-s001.zip › PHYSREP-2026-04-377-T-s05.pdf]

**Скрининговая форма**<br>Screening form<br>***ID исследователя:***<br>*(RA-ID)*<br>______________________	**ID участника:**____________________ **Имя**:____________________________<br>ID participant: Name:<br>**Дата рождения**:__________________ **Пол:** **м** **д**<br>Date of birth: Gender: b g<br>**Место исследования:**_____________ **Дата**:______________________________<br>Study sites: Date:

Критерии включения

Inclusion criteria

Если дети соответствуют следующим критериям, они могут быть включены в исследуемую группу

If the children meet the following criteria, they may be included in the study group.

Пожалуйста, отметьте каждый критерий знаком ✓

Please mark each criterion with a ✓

1. Демографические данные

Demographics

**Возраст: от 10 до 12 лет**<br>Age: 10-12 years old	
**Прожил большую часть жизни в месте проведения исследования (>50%)**<br>Lived majority of life in the study location (>50%)	
**Является учеником школы, включенной в исследование**<br>Enrolled in participating study school	

2. Документация

Documentation

**Родители или законные опекуны ребенка могут подписать форму информированного согласия.**<br>Parents or legal guardians of the child could sign the informed consent form.	

Критерии исключения

Exclusion criteria

Если есть хотя бы один из следующих критериев, ребенок должен быть исключен из исследования.

If the children meet any of the following criteria, they must be excluded from the study.

Пожалуйста, проставьте галочку (✓) рядом с любым критерием исключения.

Please mark with an ✓, if any of the exclusion criteria are met.

В случае необходимости проконсультируйтесь с Адамом или Эльвирой.

If unsure if an exclusion criterion is met, confer with either Adam or Elvira.

1. Состояние здоровья

Health status

На основании медицинского анамнеза, есть ли у ребенка хронические болезни?

Based on a brief medical history, does the child suffer from any chronic illnesses

Спросите: «*Имеются ли у вас …*»

Ask: “Do you have…”

**Бронхиальная астма**<br>Asthma	
**Сахарный диабет**<br>Diabetes	
**Эпилепсия**<br>Epilepsy	
**Заболевания щитовидной железы**<br>Thyroid disease	
**Гемофилия**<br>Hemophilia	
**Глаукома**<br>Glaucoma	
**Другие , уточнить:**<br>Others, specify:	

2. Состояние здоровья на данный момент

Current health

На основании опроса о состоянии здоровья, страдает ли ребенок острыми заболеваниями?

Based on a brief medical history, does the child suffer from any acute illnesses

Спросите: «Страдаете ли вы от...»

Ask: "Do you have..."

**Острой инфекцией верхних дыхательных путей**<br>Acute upper respiratory tract infection	
**Пневмонии**<br>Pneumonia	
**Диареи**<br>Diarrhea	
**Тяжелой инфекционной болезни**<br>Significant infection	

Другие , уточнить:

Others, specify:

3. Прием лекарственных препаратов

Medications

Спросите: «Вы сейчас используете...»

Ask: "Do you currently use any..."

**Гормональные препараты (системные и ингаляционные)**<br>Corticosteroids (systemic and inhalation)	
**Средства стимулирующего действия (амфетамин)**<br>Stimulants (amfetamines)	
**Антигистаминные**<br>Antihistamines	
**Бронходилятаторы**<br>Bronchodilators	
**Лекарства для лечения сахарного диабета**<br>Diabetes medication	
**Препараты для щитовидной железы**<br>Thyroid medication	

Другие , уточнить:

Others, specify:

4. Другие исключения

Other exclusions

**Текущее участие в других исследованиях, связанных с медицинскими вмешательствами**<br>Current participation in other studies involving health interventions	
**Не способен выполнить необходимые тесты (например, из-за физических ограничений или отсутствия взаимодействия)**<br>Unable to perform the required tests (e.g., due to physical disabilities or non-cooperation)	
**Нежелание или невозможность заполнить социально-экономическую анкету**<br>Parental non-compliance (e.g., unwilling or unable to complete the socioeconomic questionnaire)	

Другие , уточнить:

Others, specify:
