# Supplementary material for: Altitude and child development: A cross‐sectional comparative study of children in urbanized low‐altitude and rural high‐altitude settings in Kyrgyzstan
Source: Physiol Rep. 2026 Oct 12;14(19):e71132. doi: 10.14814/phy2.71132 (PMC13658936; doi:10.14814/phy2.71132)
Supplement: Supplementary file 1 [file PHY2-14-e71132-s001.zip › PHYSREP-2026-04-377-T-s01.pdf]

Supplementary data

Supplementary Questionnaire

The parent/guardian questionnaire consists of six domains:

1. Household demographics and family structure 2. Early life factors including maternal characteristics 3. Nutritional practices and 24-hour dietary recall 4. Healthcare access and vaccination status 5. Socioeconomic indicators including income, housing, utilities and water access 6. Household smoking.

Dietary diversity was inspired and adapted by previous MICS questionnaires[1], and was calculated as a simple dietary diversity count divided into six groupings: Liquid diversity score, food diversity score, overall dietary diversity, unhealthy liquid score, unhealthy food score and overall unhealthy score. The unhealthy food groupings consisted of processed meats, sweet foods and savory snacks for the solids group and soda, sports drinks and chocolate drinks for the liquids group. Each diversity group consisted of a count of each food group, and the overall scores were sums of the underlying groupings:

Liquid diversity + food diversity = overall dietary diversity.

Unhealthy liquid score + unhealthy food score = overall unhealthy score.

Supplementary data Table 1

	**Bishkek** *n* = 123		**Naryn** *n* = 143	
**Child characteristics**				
	**Value**	**%**	**Value**	**%**
**Sex**				
Male	61	49.6 %	76	53.1 %
Female	62	50.4 %	67	46.9 %
**Age**				
10	31	25.2 %	31	21.7 %
11	64	52 %	59	41.2 %
12	28	22.8 %	53	37.1 %
Age	11.5 (±0.7)		11.6 (±0.8)	
**Family characteristics**				
	**Value**	**n/N[a]**	**Value**	**n/N[a]**
Q01 Marital status		123/123		142/143
Married	114 (92.7%)		136 (95.1%)	
Single	1 (0.8%)		2 (1.4%)	
Divorced	6 (4.9%)		1 (0.7%)	
Widow/widower	2 (1.6%)		3 (2.1%)	
Other	0		0	
Q02 Who lives in the household[c]		119/123		142/143
Spouse	104 (84.6%)		133 (93%)	
Grandparents	15 (12.2%)		40 (28%)	
Relatives	8 (6.5%)		12 (8.4%)	
None/alone	6 (4.9%)		2 (1.4%)	
Other	1 (0.8%)		0 (0%)	
Q03 Number of children in family	3.7 (±1.2)	122/123	4.5 (±1.3)	143/143
**Maternal and early life characteristics**				
	**Value**	**n/N[a]**	**Value**	**n/N[a]**
Q04 Birth location	NA	122/123	NA	143/143
Q04 Birth altitude	896.3 m (±396.2)	122/123	2129.6 m (±379.5)	143/143
Q05 Maternal age[b]	28.1 yrs (±5.7)	122/123	27.8 yrs (±6.1)	141/143
Q06 Maternal height	161 cm (±6.1)	121/123	159.5 cm (±7.1)	123/143
Q06 Maternal weight	68.6 (±13.2)	121/123	68 (±11.7)	124/143
Q06 Maternal BMI	26.4 (±4.6)	121/123	26.8 (±4.8)	121/143
Q07 Skin to skin contact at birth - Yes	118 (95.9%)	123/123	138 (96.5%)	143/143
Q08 Birth weight	3349 g (±518.8)	114/123	3102.6 g (±457.2)	136/143
Q09 Primarily breast feeding the first 6 months - Yes	118 (95.9%)	123/123	134 (94.4%)	142/143
**Continued**				

**Nutritional practices**				
	**Value**	**n/N[a]**	**Value**	**n/N[a]**
Q10 Supplement use[c]		122/123		140/143
Zinc	10 (8.1%)		13 (9.1%)	
Vitamin A	16 (13%)		14 (9.8)	
Vitamin D	68 (55.3%)		107 (74.8%)	
Multivitamin	46 (37%)		35 (24.5%)	
Gulazyk	24 (19.5%)		84 (58.7%)	
Omega 3	35 (28.5%)		25 (17.5%)	
Other	10 (8.1%)		21 (14.7%)	
Q11 Use of iodized salt - Yes	120 (97.6%)	123/123	143 (100%)	143/143
Q12/13 dietary diversity	NA	123/123	NA	143/143
Food diversity score (max. 18)	5.3 (±2.5)		5.1 (±1.9)	
Liquid diversity score (max. 10)	3.7 (±1.5)		4.0 (±1.2)	
Overall diversity score (max. 28)	8.9 (±3.3)		9.1 (±2.6)	
Unhealthy food score (max. 3)	1.0 (±0.9)		0.7 (±0.7)	
Unhealthy liquid score (max. 3)	0.5 (±0.6)		0.2 (±0.5)	
Overall unhealthy score (max 6.)	1.5 (±1.1)		0.9 (±0.9)	
**Healthcare access and vaccination status**				
	**Value**	**n/N[a]**	**Value**	**n/N[a]**
Q14 Received all recommended vaccines.	114 (92.7%)	123/123	117 (81.8%)	143/143
Q15 Time to primary care	18.2 minutes (±15.4)	123/123	15.4 minutes (±8.3)	143/143
Q15 Time to secondary care	38.3 minutes (±21.0)	123/123	25.9 minutes (±21.7)	143/143
**Socioeconomic indicators**				
	**Value**	**n/N[a]**	**Value**	**n/N[a]**
Q16 Do you have electricity		123/123		143/143
Centralized grid	120 (97.6%)		142 (99.3%)	
Off-grid	2 (1.6%)		0 (0.0%)	
No electricity	1 (0.8%)		1 (0.7%)	
Other	0 (0.0%)		0 (0.0%)	
Q17 Monthly income category		106/123		140/143
Less than 10,000 SOM (< 98 €)	10 (9.4%)		38 (27.1%)	
10,001-22,000 SOM (≈ 98-216 €)	18 (17%)		42 (30%)	
22,001-30,000 SOM(≈ 216-294 €)	15 (14.2%)		28 (20%)	
30,001-40,000 SOM(≈ 294-393 €)	19 (17.9%)		11 (7.9%)	
40,001-60,000 SOM(≈ 393-589 €)	25 (23.6%)		13 (9.3%)	
60,001-100,000 SOM(≈ 589-983 €)	16 (15.1%)		7 (5.0%)	
Above 100,000 SOM(> 983 €)	3 (2.8%)		1 (0.7%)	
Q18 Do you own the house you live in		121/123		143/143
Own	98 (79.7%)		139 (97.2%)	
Rent	22 (17.9%)		3 (2.1%)	
Other	1 (0.8%)		1 (0.7%)	

Continued

Q19 Where is your water source located		123/123		143/143
In own household	107 (87%)		116 (81.1%)	
In own yard	16 (13%)		24 (16.8%)	
Elsewhere	0 (0.0%)		3 (2.1%)	
**Household smoking**				
	**Value**	**n/N[a]**	**Value**	**n/N[a]**
Q20 Does anyone smoke in the household		122/123		143/143
No one	70 (56.9%)		86 (60.1%)	
Yes, outdoors only	47 (38.2%)		54 (37.7%)	
Yes, indoors	1 (0.8%)		3 (2.1%)	
Previously smoked	4 (3.3%)		0 (0%)	
Other	0 (5)		0 (0%)	

Note: a: n/N = number of participants with available data / total participants, values presented as mean ± SD for continuous variables and n (%) for categorical variables. b: Maternal age at birth of the examined child. c: Multiple selections allowed; percentages do not sum 100%.

Supplementary data table 2

Linear regression - BMI

	Estimate [95% CI]	Std. Error	t.value	P-value
Intercept	10.08 [3.49, 16.67]	3.34	3.02	0.003
Location (ref: Lowland)	-0.24 [-1.04, 0.56]	0.41	-0.58	0.561
Gender (ref: Female)	-0.01 [-0.74, 0.71]	0.37	-0.04	0.972
Age (years)	0.09 [-0.39, 0.56]	0.24	0.36	0.721
Income category	0.14 [-0.09, 0.37]	0.12	1.18	0.239
Maternal BMI	0.16 [0.09, 0.24]	0.04	4.25	< 0.001
Birthweight	0.0006 [-0.00002, 0.001]	0.0004	1.51	0.131

Note: Complete data for all model variables was available for 215 of 266 participants (80.8%). Residual standard error: 2.637 on 208 degrees of freedom. Multiple R-squared: 0.1006, Adjusted R-squared: 0.07466 F-statistic: 3.878 on 6 and 208 DF, p-value: 0.001085.

Supplementary data table 3

Linear regression - Hemoglobin g/L

	Estimate [95% CI]	Std. Error	t.value	P-value
Intercept	139.14 [110.49, 167.79]	14.55	9.56	< 0.001
Location (ref: Lowland)	13.93 [10.57, 17.28]	1.70	8.18	< 0.001
Gender (ref: Female)	1.44 [-1.82, 4.70]	1.66	-0.87	0.385
Age (years)	-0.52 [-2.72, 1.67]	1.11	-0.47	0.639
Birthweight	-0.004 [-0.01, -0.001]	0.002	-2.44	0.015

Note: Complete data for all model variables was available for 250 of 266 participants (93.98%). Residual standard error: 12.9 on 245 degrees of freedom. Multiple R-squared: 0.2665, Adjusted R-squared: 0.2545 F-statistic: 22.25 on 4 and 245 DF, p-value: 1.097e-15.

Supplementary data table 4

Linear regression - Lung function FEV1 in liters - Pneumococcal vaccination

	Estimate [95% CI]	Std. Error	t.value	P-value
Intercept	-2.58 [-3.42, -1.74]	0.42	-6.07	< 0.001
Location (ref: Lowland)	0.17 [0.09, 0.24]	0.04	4.57	< 0.001
Gender (ref: Female)	0.09 [0.02, 0.16]	0.03	2.58	0.010
Height (cm)	0.03 [0.02, 0.03]	0.003	8.10	< 0.001
Weight (cm)	0.01 [0.008, 0.02]	0.003	4.86	< 0.001
Income category	0.03 [0.007, 0.05]	0.01	2.62	0.009
Pneumococcal complete	0.17 [0.02, 0.32]	0.07	2.27	0.024

Note: Complete data for all model variables was available for 182 of 200 participants (91%). Residual standard error: 0.2263 on 175 degrees of freedom. Multiple R-squared: 0.6157, Adjusted R-squared: 0.6026 F-statistic: 46.74 on 6 and 175 DF, p-value: < 2.2e-16.

Supplementary data table 5

Linear regression - Lung function FVC in liters - Pneumococcal vaccination

	Estimate [95% CI]	Std. Error	t.value	P-value
Intercept	-2.91 [-3.81, -2.00]	0.46	-6.34	< 0.001
Location (ref: Lowland)	0.18 [0.10, 0.26]	0.04	4.56	< 0.001
Gender (ref: Female)	0.19 [0.11, 0.26]	0.04	5.03	< 0.001
Height (cm)	0.03 [0.02, 0.04]	0.004	8.52	< 0.001
Weight (cm)	0.02 [0.01, 0.02]	0.003	5.90	< 0.001
Income category	0.02 [-0.004, 0.04]	0.01	1.64	0.103
Pneumococcal complete	0.15 [-0.01, 0.30]	0.08	1.81	0.072

Note: Complete data for all model variables was available for 182 of 200 participants (91%). Residual standard error: 0.2443 on 175 degrees of freedom. Multiple R-squared: 0.6608, Adjusted R-squared: 0.6492 F-statistic: 56.82 on 6 and 175 DF, p-value: < 2.2e-16.

Supplementary data table 6

Linear regression - Hand grip strength in kilograms

	Estimate [95% CI]	Std. Error	t.value	P-value
**Intercept**	**-26.87 [-35.09, -18.66]**	**4.17**	**-6.44**	**< 0.001**
**Location (ref: Lowland)**	**1.90 [1.22, 2.60]**	**0.35**	**5.43**	**< 0.001**
**Gender (ref: Female)**	**2.80 [2.13, 3.48]**	**0.34**	**8.19**	**< 0.001**
**Age (years)**	**1.01 [0.50, 1.52]**	**0.26**	**3.89**	**< 0.001**
**Height (cm)**	**0.19 [0.12, 0.26]**	**0.04**	**5.29**	**< 0.001**
**Weight (cm)**	**0.14 [0.08, 0.20]**	**0.03**	**4.51**	**< 0.001**

Note: Complete data for all model variables was available for 266 out of 266 (100%). Residual standard error: 2.748 on 260 degrees of freedom. Multiple R-squared: 0.5277, Adjusted R-squared: 0.5186, F-statistic: 58.1 on 5 and 260 DF, p-value: < 2.2e-16.

Supplementary data table 7

Linear regression - Birthweight - Bishkek/Naryn

	Estimate [95% CI]	Std. Error	t.value	P-value
**Intercept**	**3933.12 [2986.79, 4879.45]**	**480.46**	**8.19**	**< 0.001**
**Location (ref: Lowland)**	**-240.24 [-361.42, -119.06]**	**61.53**	**-3.91**	**< 0.001**
**Gender (ref: Female)**	**141.27 [21.06, 261.49]**	**61.03**	**2.32**	**0.021**
**Age (Years)**	**-57.06 [-138.49, 24.37]**	**41.34**	**-1.38**	**0.169**

Note: Complete data for all model variables was available for 250 out of 266 (93.98%). Residual standard error: 480.7 on 246 degrees of freedom. Multiple R-squared: 0.08893, Adjusted R-squared: 0.007782, F-statistic: 8.004 on 3 and 246 DF, p-value: 0.00004123.

Supplementary data table 8

Linear regression - Birthweight - ≥2000 meters above sea level / <2000 meters above sea level

	Estimate [95% CI]	Std. Error	t.value	P-value
Intercept	3982.77 [3019.00, 4946.54]	489.30	8.14	< 0.001
Altitude category (ref: <2000 m)	-161.46 [-284.70, -38.23]	62.56	-2.58	0.010
Gender (ref: Female)	138.30 [15.55, 261.05]	62.32	2.22	0.027
Age (Years)	-65.11 [-147.94, 17.71]	42.05	-1.55	0.123

Note: Complete data for all model variables was available for 249 out of 266 (93.6%). Residual standard error: 489.8 on 245 degrees of freedom. Multiple R-squared: 0.0581, Adjusted R-squared: 0.04656, F-statistic: 5.037 on 3 and 245 DF, p-value: 0.002098.

Supplementary data table 9

Robustness of primary associations to alternative altitude classification.

Primary model exposure: Bishkek / Naryn

Sensitivity model exposure: Birth altitude <2000 meters / ≥2000 meters

Outcome	Primary model exposure β [95% CI], P-value	Sensitivity model exposure β [95% CI], P-value
Height (cm) x Gender interaction	-3.61 [-6.74, -0.48], 0.024	-2.60 [-5.71, 0.52], 0.102
FEV1 (L)	0.17 [0.095, 0.24], < 0.001	0.15 [0.076, 0.22], < 0.001
FVC (L)	0.18 [0.10, 0.26], < 0.001	0.13 [0.05, 0.20], 0.002

1. National Statistical Committee of the Kyrgyz Republic and UNICEF. 2023 Kyrgyzstan Multiple Indicator Cluster Survey, Snapshots of Key Findings. Bishkek, Kyrgyzstan; 2024.
