# Supplementary material for: Altitude and child development: A cross‐sectional comparative study of children in urbanized low‐altitude and rural high‐altitude settings in Kyrgyzstan
Source: Physiol Rep. 2026 Oct 12;14(19):e71132. doi: 10.14814/phy2.71132 (PMC13658936; doi:10.14814/phy2.71132)
Supplement: Supplementary file 1 [file PHY2-14-e71132-s001.zip › PHYSREP-2026-04-377-T-s02.pdf]

**Карта обследуемого ребенка**<br>Case Report Form<br>***ID исследователя:***<br>*(RA-ID)*<br>____________________________	**ID участника:**____________________<br>ID participant:<br>**Дата рождения**:__________________<br>Date of birth:<br>**Место исследования:**_______________<br>Study sites:	**Имя**:_____________________________<br>Name:<br>**Пол:** **м** **д**<br>Gender: b g<br>**Дата**:________________________________<br>Date:

Антропометрические измерения

Anthropometric measurements

**Параметры**<br>Parameters	**Результаты**<br>Result	
**Рост – см**<br>Height - cm		
**Вес – кг**<br>Weight - kg		
**СОП – см**<br>MUAC - cm	**Левое:**<br>Left:	**Правое:**<br>Right:
**ОТ – см**<br>WC - cm		

Функции легких

Lung function

**Параметры**<br>Parameters	**Лучший результат в L**<br>Best in liters	**Лучший результат в %**<br>Best in %Pred	**Отклонения от нормы**<br>Abnormal values
**ОФВ1 – л**<br>FEV1 - L			*Показан на спирометре*<br>*Shown on spirometer*
**ЖЕЛ – л**<br>FVC - L			*Показан на спирометре*<br>*Shown on spirometer*
**ОФВ1/ЖЕЛ - %**<br>FEV1/FVC - %			*Показан на спирометре*<br>*Shown on spirometer*
**ПСВ - л/сек**<br>PEFR – L/min			*Показан на спирометре*<br>*Shown on spirometer*
**МОС 25-75% - л/сек**<br>FEF25-75% - L/s			*Показан на спирометре*<br>*Shown on spirometer*

Жизненно важные показатели

Vital signs

**Параметры**<br>Parameters	**Результаты**<br>Result	**Отклонения от нормы**<br>Abnormal values
**ЧСС - уд в мин**<br>Heart rate – bpm		< 40 или /or > 120
**Насыщение крови кислородом (сатурация) - %**<br>Blood oxygen saturation - %		

Сила захвата руки<br>Hand Grip strength		
	**Правая рука**<br>Right Hand	**Левая рука**<br>Left Hand
**Сила захвата руки – кг**<br>Hand Grip strength - kg		

Гемоглобин<br>Hemoglobin		
**Параметры**<br>Parameters	**Результаты**<br>Result	**Отклонения от нормы**<br>Abnormal values
**Гемоглобин ммоль/л**<br>Hemoglobin mmol/L		**Бишкек: < 6.83**<br>Bishkek: < 6.83<br>**Нарын: < 7.63**<br>Naryn: < 7.63

Примечания*
Notes

Если будут выявлены отклонения в результатах, мы свяжемся с родителями или школьной медсестрой и порекомендуем обратиться к лечащему врачу!
If anything abnormal is measured, we will contact the parents or school nurse and advise them to contact a health professional!
