# Supplementary material for: Altitude and child development: A cross‐sectional comparative study of children in urbanized low‐altitude and rural high‐altitude settings in Kyrgyzstan
Source: Physiol Rep. 2026 Oct 12;14(19):e71132. doi: 10.14814/phy2.71132 (PMC13658936; doi:10.14814/phy2.71132)
Supplement: Supplementary file 1 [file PHY2-14-e71132-s001.zip › PHYSREP-2026-04-377-T-s03.pdf]

Только для ассистента исследователя (only for RA):

ID участника: ________________ Дата: ______________
Participants ID: Date:
ID исследователя: ________________________________
(RA ID)

Лист инфомированного согласия

Consent Form

Название: Влияние высокогорья на физическое развитие и дыхательную систему ребенка: перекрестное исследование детей, проживающих в условиях низкогорья и высокогорья Кыргызстана.

Study Title: The Role of Altitude in Growth and Respiratory Health: A Cross-Sectional Study of Children in Bishkek and Mountainous Villages, Kyrgyzstan

Ответственный исследователь:
Principal Investigator:
Adam Bøcker Olsen, stud. med.
Отдел глобального здравоохранения, кафедра педиатрии и подростковой медицины в центре им. Juliane Marie, Rigshospitalet, Копенгагенский Университет, Дания
Email: adam2olsen@gmail.com
Principal Investigator: Adam Bøcker Olsen, University of Copenhagen, Denmark
Соисследователи:
Coinvestigators:
Уметалиева Мадина Айдиновна, детский невропатолог
Национальный центр охраны материнства и детства
madiumet1122@gmail.com
Контактный телефон: Тел: +996(709)795979

Введение

Introduction

Вы даете согласие на участие Вашего ребенка в исследовании, которое направлено на изучение влияния высокогорья на физическое развитие и статуса питания детей проживающих в разных регионах Кыргызстана.

You are being asked to allow your child to participate in a research study that examines the physical development and nutritional status of children in different regions of Kyrgyzstan.

Цель – изучить влияние проживания в условиях высокогорья на рост детей, пропорции тела и функциональные показатели по сравнению с детьми, проживающими в низкогорье.

The purpose of this study is to understand how living at high altitudes may impact children's growth, body composition, and functional outcomes compared to children living in the capital.

Процедуры

Procedures

После Вашего согласия мы проведем следующие обследования:

If you agree to allow your child to participate, the following will happen:

Ваш ребенок будет осмотрен специалистом. Обследование будет включать в себя:

Your child will undergo a physical examination, including measurements of:

- Измерение роста и веса ребенка.

Height and weight.

- Измерение средней окружности плеча и окружности талии

Mid-upper arm circumference and waist circumference.

Только для ассистента исследователя (only for RA):

ID участника: ________________ *Дата:*______________
Participants ID: *Date:*
*ID исследователя:*__________________________________
(RA ID)

- Оценка функции легких (измеряется с помощью портативного спирометра)

Lung function (using a portable spirometer)

- Частота сердечных сокращений и насыщения крови кислородом (измеряется с помощью портативного пульсоксиметра)

Heart rate and blood oxygen saturation (using a portable oximeter)

- Сила захвата руки (измеряется с помощью ручного динамометра)

Hand grip strength (using a hand dynamometer)

- Определение уровеня гемоглобина (измеряется с помощью портативного устройства путем небольшого прокола для взятия капиллярной крови).

Hemoglobin levels (measured with a point-of-care device using a small pinprick of blood)

Также мы просим Вас заполнить анкету о факторах, которые могут влиять на питание Вашего ребенка, таких как социально-экономическое положение, доступ к медицинской помощи, особенности питания и воздействие окружающей среды

You will also be asked to complete a questionnaire about factors that may influence your child's nutritional status, such as socioeconomic status, access to healthcare, feeding practices, and environmental exposure.

Для физического обследования потребуются 20-30 минут, его будет проводить ассистент исследователя. Опросник займет 10-15 минут.

The physical examinations will be conducted by trained researchers and will take approximately 20-30 minutes. The questionnaire will take about 10-15 minutes to complete.

Риски и дискомфорт
Risks and Discomforts

Измерения, которые мы проведем, являются неинвазивными или минимально инвазивными и несут минимальные риски. Ваш ребенок может испытать следующие ощущения:

The measurements involved in this study are non-invasive or minimally invasive and pose minimal risks. Your child may experience:

- Небольшой дискомфорт от прокола кожи во время измерения уровня гемоглобина с минимальным риском появления синяков, кровотечений или инфекции. Эти риски минимальны, так как мы строго соблюдаем все правила гигиены.

Brief discomfort from the pinprick used to measure hemoglobin, with a minor risk of bruising, bleeding, or infection, which is rare and mitigated by sterile procedures.

- Временную одышку или головокружение во время оценки функции легких (спирометрии), что является обычным явлением и обычно быстро проходит.

Temporary shortness of breath or dizziness during the lung function test, which is common and quickly resolves.

Если во время физического обследования будут выявлены какие-либо отклонения, исследователи уведомят Вас и врача Вашего ребенка.

If any abnormal findings are detected during the physical examination, the researchers will notify you and your child's healthcare provider.

Преимущества
Benefits

Проведенные нами обследования позволят лучше понять факторы, влияющие на физическое развитие детей в Кыргызстане, которые, возможно, будут использованы для разработки целевых мер по улучшению здоровья и питания детей, особенно в высокогорных районах.

While there may be no direct benefit to your child, the information gathered in this study will help researchers and policymakers better understand the factors that influence child growth and development in Kyrgyzstan. This knowledge can be used to develop targeted interventions to improve the health and nutrition of children, especially in high-altitude regions.

Только для ассистента исследователя (only for RA):

ID участника: ________________ Дата:______________
Participants ID: Date:
ID исследователя:__________________________________
(RA ID)

Конфиденциальность
Confidentiality

Вся информация о Вашем ребенке, собранная в ходе исследования, будет конфиденциальной, и доступ к данным будет иметь только исследовательская команда.
All information collected about your child during this study will be kept confidential. Only the research team will have access to the data which will be stored securely using encrypted software. Any publications or presentations resulting from this study will not identify your child by name.

Добровольное участие
Voluntary Participation

Участие в этом исследовании является полностью добровольным. Вы можете в любой момент отказаться от участия Вашего ребенка, не понеся никаких последствий. Ваше решение об отказе не повлияет на доступ Вашего ребенка к медицинской помощи или другим услугам.
Participation in this study is completely voluntary. You may choose to withdraw your child from the study at any time without penalty. Your decision whether to participate will not affect your child's access to healthcare or other services.

Контактная информация
Contact Information

Если у Вас возникнут вопросы или опасения относительно обследования, пожалуйста, свяжитесь с главным исследователем Adam Bøcker Olsen и с Исаевой Эльвирой Жумакалыевной по номеру 0709795979.
If you have any questions or concerns about the study, please contact the principal investigator, Adam Bøcker Olsen or Elvira Isaeva (phone number 0709795979)

Согласие
Consent

Подписывая эту форму, Вы даете согласие на участие Вашего ребенка в данном исследовании.
By signing this form, you agree to allow your child to participate in this research study.

ФИО ребенка: Child's Full Name

____

ФИО родителей/опекуна: Parent/Guardian full name

____

Подпись родителя/опекуна: Parent/Guardian signature____

Дата: Date____

Адрес проживания: Residential address

____

Телефон: Phone number ____

Эл.адрес: email____

Другие способы связи: Other ways to contact

____
